# Effect of L-Citrulline Supplementation on Endothelial Function and Body Composition in Post-COVID-19 Syndrome: A Randomized Clinical Trial

**DOI:** 10.3390/nu18111706

**Published:** 2026-05-27

**Authors:** Salgado-Fernández María Fernanda, Orea-Tejeda Arturo, Sierra-Vargas Martha Patricia, González-Islas Dulce, Debray-García Yazmín, Aztatzi-Aguilar Octavio Gamaliel, Galicia-Amor Susana, Hernández-López Samantha, Renteria-Serralde Getsemani, Cruz-Gijón Gabriela, Maldonado-Vargas Valeria

**Affiliations:** 1 Licenciatura en Nutriología, Facultad de Estudios Superiores Zaragoza, Universidad Nacional Autónoma de México, Ciudad de México C.P. 09230, Mexico; 2 Unidad Clínica de Investigación Cardiopulmonar y Metabólica del Servicio de Cardiología, Instituto Nacional de Enfermedades Respiratorias Ismael Cosío Villegas, Calzada de Tlalpan 4502 Col Sec XVI, Ciudad de México C.P. 14080, Mexico; oreatart@gmail.com (O.-T.A.);; 3 Departamento de Investigación en Toxicología y Medicina Ambiental, Instituto Nacional de Enfermedades Respiratorias Ismael Cosío Villegas, Ciudad de México C.P. 14080, Mexico; pat_sierra@yahoo.com (S.-V.M.P.);; 4 Laboratorio de Toxicología Inhalatoria e Integrativa, Centro de Investigación y de Estudios Avanzados del Instituto Politécnico Nacional, Ciudad de México C.P. 14330, Mexico; 5 Departamento de Rehabilitación Pulmonar, Instituto Nacional de Enfermedades Respiratorias, Ismael Cosío Villegas, Ciudad de México C.P. 14080, Mexico

**Keywords:** post-COVID-19 syndrome, endothelial function, L-citrulline, body composition, aerobic capacity

## Abstract

**Background:** Post-COVID-19 syndrome is associated with endothelial dysfunction (ED) and various sequelae, particularly in individuals who experienced critical illness during the acute phase, affecting lung function and the musculoskeletal system. L-citrulline, a nonessential amino acid, has been shown to improve endothelial function, systemic inflammation, blood pressure, and physical performance. This study aimed to assess the effects of L-citrulline supplementation on ED and body composition in patients with post-COVID-19 syndrome. **Methods:** We conducted an open-label randomized controlled clinical trial at the Instituto Nacional de Enfermedades Respiratorias in Mexico City, Mexico, from February 2021 to May 2022. Eligible subjects were adults aged ≥18 years who recovered from COVID-19 and required hospitalization during acute COVID-19. Participants were randomized 1:1 into two groups: (1) the L-citrulline group (4 g/day) and (2) the control group. The intervention lasted three months. Endothelial-related biomarkers, including endothelin-1, sE-selectin, ICAM-1, and VCAM-1, were investigated. Body composition was measured using electrical bioimpedance, and aerobic capacity was assessed with the 6 min walk test (6MWT). Treatment effects were analyzed using two-way repeated-measures ANOVA (group × time). **Results:** In total, 43 subjects participated in the study. After three months of follow-up, the intervention group showed a decrease in ICAM-1 (−32.59 ng/mg of protein; 95% CI −52.85 to −12.33 vs. −2.31 ng/mg of protein, CI 95%: −21.59 to 16.95, *p* = 0.034) and an increase in 6MWT (141.2 m; 95% CI: 98.40 to 184 vs. 67.70 m, CI 95%: 30.62 to 104.78, *p* = 0.011) compared with the control group. No differences in body composition were observed between the groups at follow-up. **Conclusions:** L-citrulline supplementation for three months decreased ICAM-1 and increased 6MWT.

## 1. Introduction

Post-COVID-19 syndrome is defined as the persistence of symptoms and/or delayed remission of complications from SARS-CoV2 infection occurring more than four weeks after the onset of symptoms [1]. Due to a severe inflammatory response during the acute phase of the disease, alterations in endothelial function occur both during the acute phase and in the post-COVID-19 period [2]. ED has emerged as a primary determinant of macro- and microvascular dysfunction [2]. It is characterized by the reduction in endothelial nitric oxide synthase, resulting in reduced nitric oxide (NO^•^) availability [3]. NO^•^ plays an important role in vascular homeostasis due to its antithrombotic and anti-atherogenic properties. Consequently, changes in endothelial function contribute to tissue lesions [2].

Cardiovascular sequelae have been reported to persist after recovery, primarily focusing on cardiac lesions [4] and vascular endothelial damage [5]. This phenomenon is attributed to an inflammatory response that leads to excessive endothelial activation and compromises its homeostatic capacity [6]. In individuals who have recovered from COVID-19, elevated concentrations of markers indicative of endothelial damage have been observed, including endothelin-1 (ET-1) [7], a potent vasoconstrictor that is expressed under conditions associated with endothelial cell injury [8], has been linked to the development of pulmonary damage and is a possible marker of poor prognosis [9,10]. Additionally, there is an increased level of adhesion molecules such as E-selectin, intercellular adhesion molecule 1 (ICAM-1), and vascular cell adhesion molecule 1 (VCAM-1), which are expressed in immune and epithelial cells as part of the inflammatory response [11] and are considered markers of endothelial injury and/or cellular activation [12]. These changes are associated with the upregulation of inflammatory cytokine production, including interleukin-1 and tumor necrosis factor-alpha (TNF-α), potentially contributing to a hypercoagulable state [13].

The hyperinflammatory immune response to COVID-19 is also linked to metabolic changes that occur, involving adipogenesis and lipolysis pathways, as well as with Homeostatic Model Assessment of Insulin Resistance (HOMA-IR) levels, leading to increased immune dysfunction and chronic inflammatory conditions. L-citrulline, a water-soluble nonessential alpha-amino acid and a precursor of L-arginine, plays a crucial role in enhancing NO^•^ bioavailability. It also serves as a substrate involved in regulating vascular tone, platelet aggregation, and leukocyte adhesion [14,15]. Oral supplementation with L-citrulline has demonstrated positive effects on the antihypertensive and vasodilatory capacities of the endothelium, enhancing the flow-mediated dilatation (FMD) and plasma L-arginine/asymmetric dimethylarginine (ADMA) ratio by increasing NO^•^ biosynthesis [14,15]. Additionally, L-citrulline supplementation has been associated with the modulation of chronic low-grade inflammation, reducing concentrations of interleukin-6, TNF-α, and C-reactive protein, while preserving anti-inflammatory cytokines (such as interleukin-10) and NO^•^ production [16]. In the short term, it may also contribute to arterial stiffness reduction [17].

On the other hand, there is a connection between endothelial function and body composition; an increase in visceral fat is linked to a decline in FMD [18]. Additionally, higher ADMA levels are associated with lower muscle strength and a greater prevalence of sarcopenia [19]. Regarding L-citrulline supplementation, it has been found that, when combined with exercise, it decreases body fat [20] and improves muscle strength [21]. Persistent endothelial dysfunction, reduced physical performance, and musculoskeletal alterations have been described in patients with post-COVID-19 syndrome. Given the role of L-citrulline as a precursor of NO^•^ synthesis, its supplementation may potentially influence endothelial-related pathways and functional recovery. However, evidence evaluating the effects of L-citrulline supplementation on endothelial biomarkers and body composition in patients with post-COVID-19 syndrome remains limited. The primary objective of this research was to evaluate the impact of L-citrulline supplementation on endothelial function, with secondary objectives to assess the effect of this supplementation on body composition and the six-minute walk test (6MWT) in patients recovered from COVID-19.

## 2. Materials and Methods

### 2.1. Study Design

An open-label, randomized controlled clinical trial was conducted at the Post-COVID Clinic of the Instituto Nacional de Enfermedades Respiratorias Ismael Cosío Villegas in Mexico City, México. The clinic provides specialized follow-up care for patients with persistent sequelae after severe COVID-19 that required hospitalization during the acute phase of the disease. The study was carried out between February 2021 and May 2022, in accordance with the Declaration of Helsinki, and was approved by the Institutional Review Board and Ethics Committee (approval number C71-20, 9 November 2020). The study is registered at https://clinicaltrials.gov/ (NCT07544186). This study followed the Consolidated Standards of Reporting Trials (CONSORT). All participants voluntarily provided written informed consent prior to enrollment.

### 2.2. Study Population

**Figure 1:**
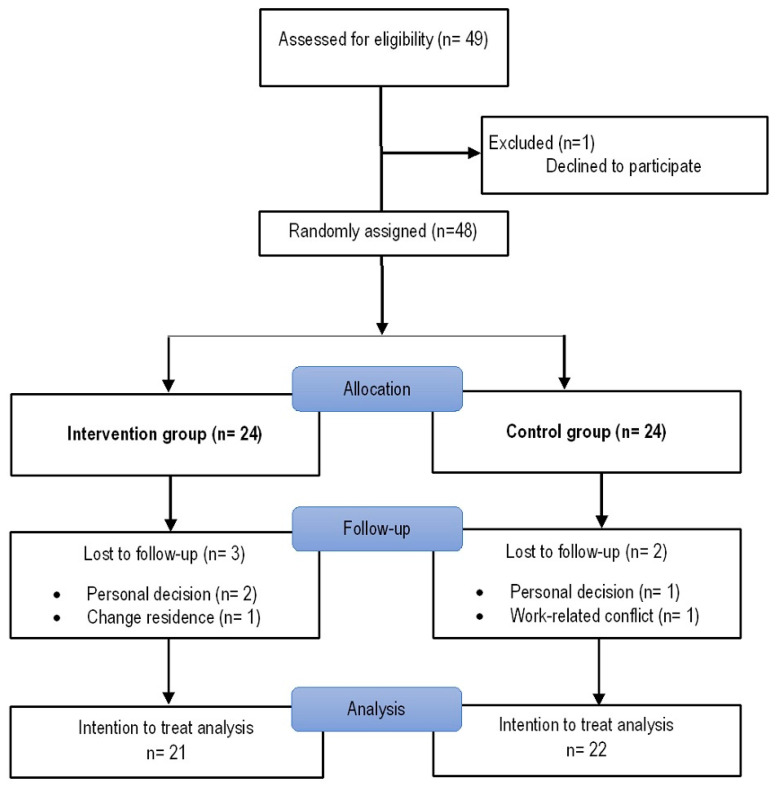
Study flow diagram.

This study focused on individuals who recovered from COVID-19 and required hospitalization during acute infection. Eligible subjects were adults aged 18 years or older who had recovered from COVID-19, with a confirmed diagnosis of COVID-19 by RT-PCR testing during the acute phase, and who had been hospitalized due to moderate to severe COVID-19, with a PaO_2_/FiO_2_ ratio < 300 (arterial partial pressure of oxygen/fraction of inspired oxygen). Three months after hospital discharge, patients agreed to participate and signed the informed consent form. Subjects with a glomerular filtration rate < 30 mL/min/1.73 m^2^ were excluded. Patients who had been hospitalized at our institution were contacted about 2 to 3 months after discharge for a thorough assessment at the post-COVID-19 outpatient clinic. Through the nutritional service, potentially eligible participants were approached and invited to enroll in the study protocol. At the baseline evaluation, subjects who met the inclusion criteria and agreed to participate provided written informed consent before being randomly assigned to either the intervention group or the control group (Figure 1).

### 2.3. Randomization

Randomization was performed using Stata version 14 (Stata Corp., College Station, TX, USA). A computer-generated random allocation sequence with permuted blocks was used to ensure a balanced 1:1 allocation between the intervention and control groups.

### 2.4. Allocation and Interventions

Subjects were randomly assigned using a sequence with a 1:1 allocation ratio, including multiple block sizes, to one of two groups. (1) The intervention group received 4 g per day of L-citrulline, administered as a powder sachet that participants dissolved in water and consumed as a single daily dose. Participants received a total of 90 sachets for a three-month intervention period, along with normocaloric nutritional treatment (50% to 55% carbohydrates, 20% to 25% protein, and 20% to 25% lipids) and usual medical care. L-citrulline supplement adherence was defined as consumption of at least 80% of the assigned sachets. Additionally, participants received a diary to record the days on which the supplement was consumed. (2) The control group received normocaloric nutritional treatment (50% to 55% carbohydrates, 20% to 25% protein, and 20% to 25% lipids) and usual medical care. The intervention lasted for three months.

All assessments, including endothelial biomarkers, anthropometry, body composition, handgrip strength, and 6MWT, were conducted by trained personnel who were not involved in the intervention procedures.

### 2.5. Endothelium Biomarkers

Fasting blood samples were collected in the morning. Samples were centrifuged to separate the plasma fraction, which was stored at −80 °C until biomarker measurements. All samples were thawed and immediately analyzed in a single freeze–thaw cycle before use.

NO^•^ was analyzed with the immunosorbent assay (ELISA) kit (KGE001, R&D Systems, Inc., Minneapolis, MN, USA). This method detects the formation of nitrite at 540 nm during the enzymatic conversion of nitrate to nitrite by nitrate reductase. A standard curve ranging from 3.13 to 200 μmol/L was generated using a four-parameter logistic (4-PL) curve fit. The minimum detectable dose (MDD) was 0.25 μmol/L. The results were expressed in μmol/L. Human sE-Selectin/CD62E (Cat. SSLE00) and ET-1 (SET100) levels were measured using an ELISA kit (R&D Systems, Inc., Minneapolis, MN, USA). The measurements were performed following the specifications of each kit. E-Selectin concentrations were expressed in ng/mL. Total plasma protein was measured using the Lowry method [22], with absorbance measured at 550 nm using bovine serum albumin (BSA, Sigma) as a standard.

### 2.6. Anthropometry

Weight and height were measured according to the techniques described in the manual reference for anthropometric standardization. Subjects wore light clothing and were barefoot during measurements [23].

### 2.7. Body Composition

Body composition was measured using whole-body electrical bioimpedance with RJL Systems Quantum single-frequency (Quantum IV, RJL Systems, Clinton Township, Michigan, USA). The standardized technique was used [24]; subjects were fasting, had not exercised for eight hours, and had not consumed alcohol for 12 h before the study. The participants were placed in a supine position, with the arms separated from the trunk at about 30° and the legs separated at about 45°. The area was cleaned with alcohol, and electrodes were placed on the ipsilateral hand and foot after skin antisepsis. Patients were required to fast for at least two hours before the test and abstain from physical exercise, saunas, and alcohol for 12 h prior. All measurements were conducted in a comfortable area, free of air currents, by the same operator. Phase angle (PhA) was calculated using the following equation: PhA (degrees) = arctan (Xc/R)·(180/π) [25]. The appendicular skeletal muscle mass index (ASMMI) was assessed according to Sergi’s equation [26]:

ASMM (kg/m^2^) = [−3.94 + (0.227 ∗ Height^2^ (cm)/Resistance) +  (0.095 ∗ Weight) + (1.384 ∗ Sex) + (0.064 ∗ Reactance)]/Height (m^2^)

### 2.8. Handgrip Strength

Handgrip strength was measured using a mechanical Smedley Hand Dynamometer (Stoelting, Wood Dale, UK), adjustable to fit the hand width. Subjects stood with arms outstretched parallel to the torso, then took the dynamometer and exerted maximum force with each hand without support. The measurement was repeated three times alternately in the dominant hand, with a 1 min interval to prevent fatigue. The highest value was recorded in kilograms [27].

### 2.9. Aerobic Capacity

Aerobic capacity was evaluated using a 6MWT performed according to American Thoracic Society standards [28].

### 2.10. Dietary Intake

Dietary intake was measured using a 24 h record. We asked participants about food and beverage consumption from the previous day [29]. Food Processor software version 11.1 was used to analyze energy and macronutrient intake.

### 2.11. Statistical Analysis

Sample size was estimated a priori using G*Power software (version 3.1.9.7, Düsseldorf, Germany) based on data from a clinical trial evaluating the effects of taurine supplementation (3000 mg daily for 8 weeks) versus starch placebo on ICAM-1 levels [30]. The sample size calculation was based on an expected difference in the primary endothelial-related biomarker outcome between the study groups. A total of 28 participants were estimated to be required to detect a group difference with 95% power at α = 0.05, assuming a 20% loss to follow-up. Data analyses were performed using the commercially available package Stata version 14 (Stata Corp., College Station, TX, USA). Qualitative variables were presented as frequencies and percentages. The Shapiro–Wilk test was employed to assess the normality of continuous variables. Normally distributed continuous variables were expressed as mean and standard deviation, whereas non-normally distributed variables were presented as median and percentiles 25 to 75. A comparison among the different study groups was analyzed using the chi-square test for qualitative variables and Student’s *t*-test or Mann–Whitney U test for continuous variables.

The effect of L-citrulline supplementation after three months of follow-up was assessed using a modified intention-to-treat analysis, which included all participants with available follow-up data according to their randomized group allocation, regardless of adherence to the intervention. Treatment effects were examined using two-way repeated-measures ANOVA (group × time) and additionally adjusted for variables with *p*-values < 0.10, such as E-Selectin and hospital stay, using bivariate analysis. A *p* < 0.05 was considered statistically significant.

## 3. Results

Forty-three patients were analyzed; the mean age was 53.65 ± 13.96 years; 53.49% were male; 53.49% had obesity; 34.88% had hypertension; and 30.23% had diabetes. Additionally, 65.12% required mechanical ventilation during hospitalization. Participants in the intervention group exhibited significantly lower E-selectin levels (72.31 ± 13.07 vs. 82.55 ± 12.77; *p* = 0.015) than those in the control group. There were no statistically significant differences in the remaining variables between the study groups (Table 1).

Regarding the effect of L-citrulline supplementation, adherence to the supplementation was 85%; after three months of follow-up, the intervention group demonstrated a reduction in ICAM-1 levels (−32.59 ng/mg of protein, CI 95%: −52.85 to −12.33; *p* = 0.001), while the control group showed no change (−2.31 ng/mg of protein, CI 95%: −21.59 to 16.95; *p* = 0.813). A difference was observed between the study groups throughout the follow-up period.

Regarding body composition, the intervention group showed an increase in PhA (0.49°, CI 95%: −0.01 to 0.97; *p* = 0.043). In contrast, the control group showed no changes (−0.12°, CI 95%: −0.59 to 0.33; *p* = 0.583); however, no differences between the study groups were observed (*p* = 0.067). With respect to handgrip strength, both the intervention group (2.39 kg, CI 95%: 0.63 to 4.15; *p* = 0.007) and the control group (2.35 kg, CI 95%: 0.59 to 4.11; *p* = 0.008) showed an increase in handgrip strength, but no significant difference between the study groups was observed.

**Figure 2:**
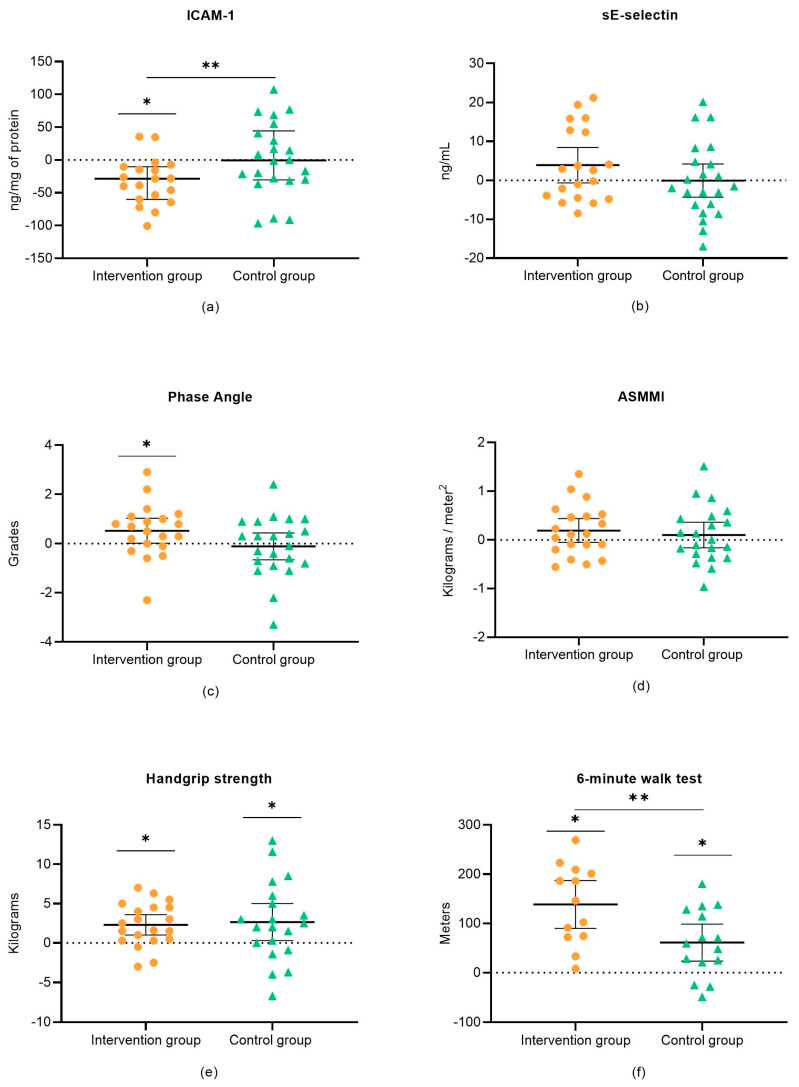
Changes in biomarkers, body composition, and muscle, and the six-minute walk test in the intervention and control groups after 3 months of follow-up. (**a**): Intercellular adhesion molecule 1; (**b**): sE-selectin; (**c**): Phase angle; (**d**): Appendicular skeletal muscle mass index; (**e**): Handgrip strength; (**f**): 6-min walk test. * *p*-value between a baseline and a 3-month ** *p* < 0.05 vs. control group.

Regarding the 6MWT, both the intervention group (141.2 m, CI 95%: 98.40 to 184.00; *p* < 0.001) and the control group (67.70 m, CI 95%: 30.62 to 104.78; *p* < 0.001) showed improvement, with greater improvement in the intervention group (*p* = 0.011) (Table 2 and Figure 2).

Concerning dietary intake, no modifications were observed during the follow-up period in either the intervention or control group (Table 2).

## 4. Discussion

The main finding of our study was that supplementation with L-citrulline was associated with decreased ICAM-1 levels and improved aerobic capacity. ICAM-1 regulates leukocyte recruitment and is primarily expressed during the inflammatory response [11]. ICAM-1 on endothelial cells plays an important role in the migration of activated leukocytes to sites of inflammation [11]. In COVID-19 survivors, a persistent hyperinflammatory state is known to occur, promoting increased secretion of inflammatory mediators [1]. ED has been associated with vascular inflammation and elevated expression of adhesion molecules, including ICAM-1. During acute COVID-19, SARS-CoV-2 may infiltrate endothelial cells [31], a phenomenon associated with disease severity and poor prognosis, while the expression of inflammatory and endothelial activation markers may persist after recovery [32].

Within this framework, ED contributes to atherogenic processes by promoting the production of adhesion molecules that recruit inflammatory monocytes to the vessel wall, leading to their overexpression [33]. Moreover, in COVID-19-recovered patients, elevated levels of ICAM-1 were observed five weeks after the initial diagnosis, indicating it as a marker of late infection sequelae [34]. However, our findings should be interpreted with caution, as only ICAM-1 showed a significant group × time effect, while the other endothelial biomarkers did not significantly differ between groups. Additionally, recent evidence indicates that circulating adhesion molecules such as ICAM-1, VCAM-1, and E-selectin may not directly correlate with physiological measures of macrovascular and microvascular endothelial function [35]. In our study, we observed a decrease in ICAM-1; similar results were observed in a clinical trial conducted in hypertensive postmenopausal women, in which supplementation with 10 g/day of L-citrulline improved FMD [36]. L-citrulline is a precursor of L-arginine, which enhances NO^•^ bioavailability, a substrate involved in regulating vascular tone and platelet aggregation [37]. Oral supplementation with L-citrulline has demonstrated positive effects on endothelial vasodilatory capacity, likely due to enhanced NO^•^ biosynthesis [14]. Moreover, alterations in endothelial function have been associated with increased vascular smooth muscle tone through reduced production of vasodilatory substances, such as NO^•^ [38].

Regarding supplementation with other amino acids, Tosato et al. demonstrated that supplementation with 1.66 g of L-arginine plus 500 mg of vitamin C for 28 days in long COVID subjects improved aerobic capacity and endothelial function measured by FMD [39]. Furthermore, previous studies have reported reductions in ICAM-1 levels following nutritional interventions associated with modulation of inflammation and endothelial activation. Similar to our study, Schwingshackl and Hoffmann observed that greater adherence to the Mediterranean diet pattern was associated with a significant decrease in ICAM-1 levels (−23.73 ng/mL, 95% CI −41.24, −6.22; *p* = 0.008) [40]. This effect has been attributed to increased intake of monounsaturated fatty acids and reduced saturated fatty acid consumption, both of which may contribute to decreased inflammatory responses [40]. Likewise, supplementation with 2000 IU/day of vitamin D in patients with hypertension resulted in a significant reduction in ICAM-1 levels compared with placebo, suggesting a potential role in modulating inflammatory balance [41]. In this context, L-citrulline, as a precursor of L-arginine and NO^•^ synthesis, may similarly modulate endothelial inflammatory activation and adhesion molecule expression.

PhA has been described as an indicator of nutritional status, a clinical condition, and a prognostic marker [42], with a close relationship to hydration status and muscle markers [43]. In patients with post-acute COVID-19, lower PhA values were associated with reduced handgrip strength, suggesting poor nutritional status [44]. In the present study, although a slight increase in PhA was observed after three months of follow-up, no statistically significant differences were identified between groups. Similarly, no significant between-group differences were observed in body composition parameters. These findings may suggest that isolated L-citrulline supplementation was insufficient to induce measurable changes in body composition or cellular integrity markers during the study period.

In our study, both the intervention and control groups showed improvements in handgrip strength and aerobic capacity. However, the intervention group demonstrated a significant improvement in aerobic capacity. These findings may partially reflect the natural recovery process after severe COVID-19, including progressive improvement in physical conditioning, resolution of systemic inflammation, and recovery from prolonged hospitalization and physical deconditioning. In addition, all participants received nutritional treatment and usual medical care, which may have contributed to functional improvement independently of L-citrulline supplementation.

The six-minute walk test has been described as an indicator of functional and clinical status, as well as a useful tool for obtaining relevant diagnostic and prognostic information [45]. Reduced muscle strength has been identified as a strong predictor of prolonged hospital stays and decreased functional capacity, thereby limiting the ability to perform activities of daily living and influencing prognosis in diverse populations [46,47]. Several mechanisms may explain the improvement in muscle performance, L-citrulline has been shown to increase plasma levels of L-arginine, which is the substrate for the synthesis of NO^•^. Additionally, it has been observed to increase NOS synthesis; thus, L-citrulline may indirectly enhance NO^•^ production and restore it when compromised. As a result, this increases blood flow and improves oxygen and energy supply to activate muscles, thereby enhancing muscle performance [48,49]. However, Hickner et al. observed a reduction in treadmill time to exhaustion with L-citrulline supplementation [50]. (2) L-citrulline plays an important role in nitrogen homeostasis. It is a component of the urea cycle in the liver, where L-arginine is produced from L-citrulline and catabolized into ornithine and urea. Since urea is the main route for removing ammonium, which has been linked to muscle fatigue, supplementation with L-citrulline may improve ammonium homeostasis and consequently enhance muscle functionality [51,52].

Similarly, Bailey et al. [53] demonstrated that short-term oral supplementation with 6 g of L-citrulline for seven days improved muscle oxygenation during high-intensity exercise in healthy adults by increasing availability and distribution within the muscle microvasculature, thereby enhancing muscle performance. Furthermore, the benefits of L-citrulline may be mediated by improved peripheral vasodilation/perfusion, promoting muscle oxygen utilization. This effect is likely because of increased NO^•^ availability in skeletal muscles, which may contribute to muscle function and mass [54].

Moreover, in our experience, L-citrulline significantly improved left ventricular ejection fraction, endothelial function, and functional class. Citrulline is an important co-adjuvant in the treatment of compensated systolic heart failure patients [55].

COVID-19 disease is characterized by alterations not only in the lungs but also in other tissues such as the endothelium and musculoskeletal system. It has been observed that endothelial damage may persist even after patients have recovered [32].

Conversely, ET-1 is recognized as a potent vasoconstrictor in the cardiovascular system and plays a central role in vascular tone regulation [56], whereas L-citrulline enhances NO production and promotes vasodilation. Despite these potentially opposing physiological effects, no significant changes in ET-1 levels were observed in our study. To date, evidence evaluating the effects of L-citrulline supplementation on circulating ET-1 levels in clinical trials remains limited.

Nitrite is a stable biomarker of NO. Plasma nitrite reflects endothelial nitric oxide synthase activity and has emerged as a diagnostic marker of both endothelial function and cardiovascular diseases [57]. Although a clinical trial conducted on subjects with type 2 diabetes demonstrated that supplementation with 3 g/day of L-citrulline increased serum nitrate/nitrite ratio levels compared with placebo [58], no significant differences in nitrite levels were observed in our study. These discrepant findings may be attributable to differences in clinical populations, baseline endothelial status, or persistent inflammatory or vascular alterations present in post-COVID patients. The principal strengths of our study include the randomized controlled clinical trial design and the study groups according to the intention-to-treat principle, whereby all subjects were analyzed according to their random group assignment regardless of patient compliance. This approach prevents overestimating the effects of therapy in the study, and the intervention is likely more effective in patients who adhere to L-citrulline supplementation [59]. Nevertheless, several limitations should be acknowledged, for instance, the single-center study, with a small sample size, and short follow-up. Furthermore, the sample size calculation was based on data from a previous nutritional supplementation study conducted in a different clinical population, because there were no prior studies evaluating L-citrulline supplementation in post-COVID-19 patients. Additionally, because of the open-label design of the study, participants and treating investigators could not be blinded, which might have introduced performance bias. However, outcome assessors were blinded to treatment allocation to reduce measurement bias during functional and clinical evaluations. Another limitation was the absence of an evaluation of physical activity levels throughout the follow-up period. This could have served as a confounding factor affecting muscle strength, body composition, and functional capacity.

## 5. Conclusions

Our study showed that supplementation with L-citrulline for three-months reduced ICAM-1 concentrations, as well as increasing aerobic capacity in patients with post-COVID-19 syndrome.

## Figures and Tables

**Table 1:** Baseline characteristics according to study group.

	Total	Intervention Group	Control Group	*p*-Value
	*n* = 43	*n* = 21	*n* = 22	
Men, *n* (%)	23 (53.49)	14 (66.67)	9 (40.91)	0.129
Age, (years)	53.65 ± 13.96	55.38 ± 11.16	52 ± 16.29	0.434
Comorbidities, *n* (%)				
Obesity	23 (53.49)	11 (52.38)	12 (54.55)	0.887
Hypertension	15 (34.88)	8 (38.10)	7 (31.82)	0.666
Diabetes	13 (30.23)	7 (33.33)	6 (27.27)	0.665
Body composition and physical function				
Weight, (kg)	85.60 ± 19.03	87.67 ± 19.44	83.63 ± 18.87	0.493
Height, (cm)	164.18 ± 8.95	165.61± 6.74	162.81± 10.63	0.307
BMI, (kg/m^2^)	31.63 ± 6.03	31.88 ± 6.63	31.39 ± 5.55	0.793
Phase angle, (°)	6.33 ± 0.85	6.27 ± 0.72	6.4 ± 0.96	0.611
ASMMI, (kg/m^2^)	6.89 ± 1.08	7.12 ± 1.00	6.69 ± 1.12	0.200
Handgrip strength, (kg)	24.85 ± 9.61	25.76 ± 9.36	23.95 ± 10.01	0.548
Six-minute walk test, (m)	428.34 ± 93.34	421.14 ± 73.66	433.94 ± 108.00	0.707
Endothelial function markers				
E-Selectin, (ng/mL)	77.80 ± 13.75	72.31 ± 13.07	82.55 ± 12.77	0.015
Endothelin-1, (pg/mL)	1.35 ± 0.54	1.42 ± 0.52	1.28 ± 0.57	0.447
Nitrite, (pg/mL)	15.79 ± 4.78	16.04 ± 4.32	15.57 ± 5.24	0.761
ICAM-1, ng/mg of protein	305.64 ± 89.24	305.61 ± 93.76	305.67 ± 87.38	0.998
VCAM-1, ng/mg of protein	1377.28 ± 448.78	1415.70 ± 477.94	1344.10 ± 430.51	0.616
Hospital parameters				
Hospital stay, days	21.5 (11–41)	18 (11–26)	30 (11–47)	0.064
Mechanical ventilation, *n* (%)	28 (65.12)	14 (66.67)	14 (63.64)	0.835
PaO_2_/FiO_2_	160.51 ± 78.35	153.37 ± 89.99	168.05 ± 65.63	0.576
Pulmonary function				
FEV_1_, (% of predicted value)	86.01 ± 18.21	86.51 ± 19.76	85.48 ± 17.04	0.874
FVC, (% of predicted value)	80.75 ± 17.63	80.43 ± 19.22	81.09 ± 16.40	0.916
FEV_1_/FVC	0.81± 0.07	0.81 ± 0.05	0.81 ± 0.09	0.832
Treatment				
Antihypertensive, *n* (%)	13 (30.23)	7 (33.33)	6 (27.27)	0.665
Glucose-lowering, *n* (%)	16 (37.21)	8 (38.10)	8 (36.36)	0.907
Lipid-lowering, *n* (%)	6 (13.95)	2 (9.52)	4 (18.18)	0.413

BMI: body mass index; ASMMI: appendicular skeletal muscle mass index; ICAM-1: intercellular adhesion molecule 1; VCAM-1: vascular cell adhesion molecule-1; PaO_2_/FiO_2_: arterial partial pressure of oxygen/fraction of inspired oxygen ratio; FEV_1_: forced expiratory volume in 1 s; FVC: forced vital capacity; FEV_1_/FVC: forced expiratory volume in 1 s/forced vital capacity ratio.

**Table 2:** Effect of L-citrulline supplementation on endothelial biomarkers, body composition and physical function.

	Group	Pre	Post	Δ (CI 95%)	*p*-Value Within Group	*p*-Value Group × Time
Endothelial biomarkers						
sE-selectin, (ng/mL)	IG	72.31 ± 13.07	76.21 ± 11.89	3.89 (−0.18 to 7.97)	0.061	0.182
	CG	82.55 ± 12.77	82.46 ± 13.59	0.05 (−3.82 to 3.93)	0.977	
Endothelin-1, (pg/mL)	IG	1.27 [0.93–1.91]	1.12 [0.87–1.57]	−0.08 (−0.37 to 0.20)	0.557	0.443
	CG	1.07 [0.89–1.41]	1.27 [0.99–1.61]	0.06 (−0.37 to 0.20)	0.620	
Nitrite, (pg/mL)	IG	15.07 [13.3–19.1]	14.21 [12.7–16.2]	0.52 (−10.64 to 11.69)	0.926	0.436
	CG	14.5 [13.1−15.3]	14.06 [13.3−16.5]	6.64 (−3.97 to 17.26)	0.220	
ICAM-1, (ng/mg of protein)	IG	305.61 ± 93.76	273.02 ± 86.03	−32.59 (−52.85 to −12.33)	0.001	0.034
	CG	305.67 ± 87.38	306.95 ± 71.45	−2.31 (−21.59 to 16.95)	0.813	
VCAM-1,(ng/mg of protein)	IG	1415.7 ± 477.94	1399.47 ± 421.85	−16.22 (−182.9 to 150.5)	0.848	0.922
	CG	1344.10 ± 430.51	1358.22 ± 395.14	−4.7 (−163.3 to 153.8)	0.952	
Body composition						
Weight, (kg)	IG	87.67 ± 19.44	86.73 ± 17.86	−0.94 (−3.30 to 1.4)	0.134	0.110
	CG	83.63 ± 18.87	85.25 ± 18.95	1.71 (−0.52 to 3.95)	0.433	
Phase angle, (°)	IG	6.27 ± 0.72	6.79 ± 0.88	0.49 (−0.01 to 0.97)	0.043	0.067
	CG	6.4 ± 0.96	6.29 ± 1.06	−0.12 (−0.59 to 0.33)	0.583	
Total body water, (%)	IG	48.37 ± 8.60	48.67 ± 9.41	0.97 (−2.78 to 1.92)	0.427	0.412
	CG	45.66 ± 9.33	45.36 ± 10.29	−0.43 (−2.78 to 1.92)	0.719	
Extracellular water, (%)	IG	12.59 ± 1.65	12.68 ± 1.71	0.21 (−0.32 to 0.756)	0.441	0.300
	CG	12.33 ± 1.79	12.20 ± 1.83	−0.18 (−0.71 to 0.34)	0.486	
Intracellular water, (%)	IG	35.77 ± 7.14	35.99 ± 7.88	0.21 (−1.90 to 2.34)	0.832	0.778
	CG	33.32 ± 7.74	33.16 ± 8.59	−0.16 (−2.01 to 1.68)	0.854	
Body fat mass, (%)	IG	28.96 ± 8.44	28.1 ± 6.60	−1.89 (−4.22 to 0.43)	0.110	0.606
	CG	35.66 ± 9.82	34.48 ± 10.48	−0.95 (−3.68 to 1.77)	0.494	
ASMMI, (kg/m^2^)	IG	7.12 ± 1.00	7.31 ± 1.24	0.52 (−0.07 to 1.12)	0.087	0.584
	CG	6.68 ± 1.15	6.79 ± 1.29	0.16 (−0.41 to 0.73)	0.5836	
Handgrip strength, (kg)	IG	26.15 ± 9.42	28.45 ± 10.08	2.39 (0.63 to 4.15)	0.007	0.973
	CG	24.4 ± 10.05	27.05 ± 9.80	2.35 (0.59 to 4.11)	0.008	
Six-minute walk test, (m)	IG	414.38 ± 72.00	552.84 ± 65.29	141.2 (98.40 to 184.0)	<0.001	0.011
	CG	440.13 ± 96.27	501.13 ± 27.61	67.70 (30.62 to 104.78)	<0.001	
Nutrient intake						
Energy, (kcal)	IG	1521.57 ± 505.87	1412.92 ± 406.36	−216.0 (−561.1 to 129.1)	0.219	0.361
	CG	1521.19 ± 778.13	1537.78 ± 467.83	7.21 (−324.9 to 339.4)	0.966	
Protein, (%)	IG	19.09 ± 3.73	20.23 ± 4.92	1.61 (−1.58 to 4.81)	0.322	0.127
	CG	20.12 ± 8.33	17.98 ± 4.60	−1.84 (−4.92 to 1.23)	0.241	
Carbohydrates, (%)	IG	49.85 ± 11.77	45.78 ± 11.39	−3.81 (−11.55 to 3.91)	0.333	0.124
	CG	48.82 ± 11.39	53.10 ± 14.51	4.61 (−2.83 to 12.06)	0.224	
Lipids, (%)	IG	32.53 ± 11.65	35.61 ± 10.30	2.43 (−7.78 to 5.82)	0.500	0.496
	CG	32.44 ± 12.08	31.77 ± 11.68	−0.97 (−7.78 to 5.82)	0.778	
Saturated fat, (%)	IG	11.3 ± 5.42	10.92 ± 3.84	−0.85 (−3.67 to 1.96)	0.552	0.372
	CG	10.54 ± 4.70	11.22 ± 4.26	0.93 (−1.78 to 3.64)	0.502	
Fiber, (g)	IG	19.53 ± 10.61	21.93 ± 6.27	0.63 (−4.60 to 5.86)	0.813	0.816
	CG	18.88 ± 6.56	18.66 ± 9.25	−0.23 (−5.27 to 4.81)	0.928	

ICAM-1: intercellular adhesion molecule 1; VCAM-1: vascular cell adhesion molecule-1; ASMMI: appendicular skeletal muscle mass index.

## Data Availability

The data presented in this study are available on request from the corresponding author due to ethical reasons.

## Abbreviations

ADMA	Asymmetric Dimethylarginine
ASMMI	Appendicular Skeletal Muscle Mass Index
CONSORT	Consolidated Standards of Reporting Trials
ED	Endothelial Dysfunction
eNOS	Endothelial Nitric Oxide Synthase
ET-1	Endothelin-1
FMD	Flow-mediated Dilatation
HOMA-IR	Homeostatic Model Assessment of Insulin Resistance
ICAM-1	Intercellular Adhesion Molecule 1
NO	Nitric Oxide
TNF-α	Tumor Necrosis Factor-Alpha
VCAM-1	Vascular Cell Adhesion Molecule-1
6MWT	Six-Minute Walk Test
